# Influence of a transverse static magnetic field on the orientation and peritectic reaction of Cu-10.5 at.% Sn peritectic alloy

**DOI:** 10.1038/s41598-018-28888-8

**Published:** 2018-07-13

**Authors:** Zhenyuan Lu, Yves Fautrelle, Zhongming Ren, Xi Li

**Affiliations:** 1 State Key Laboratory of Advanced Special Steels, Shanghai University, Shanghai, 200072 P. R. China; 2 SIMAP-EPM-Madylam/G-INP/CNRS, Phelma, 38402 Saint Martin d’Heres Cedex, France

## Abstract

Peritectic alloy Cu-10.5 at.% Sn was directionally solidified at various growth speeds under a transverse static magnetic field. The experimental results indicated that the magnetic field caused the deformation of macroscopic interface morphology, the crystal orientation of primary phase along solidification direction, and the occurrence of peritectic reaction. The numerical simulations showed that the application of the magnetic field induced the formation of a unidirectional thermoelectric magnetic convection (TEMC), which modified solute transport in the liquid phase thereby enriching the solute concentration both at the sample and tri-junction scales. The modification of solidification structures under the magnetic field should be attributed to TEMC driven heat transfer and solute transport.

## Introduction

Peritectic reaction is commonly observed in many binary alloys, such as Fe-based (Fe-C, Fe-Ni), Cu-based (Cu-Zn, Cu-Sn) and Al-based (Al-Ti) alloys^1^. In peritectic metallic system, various solidification structures have been experimentally observed during directional solidification^2–5^. A large number of numerical simulations and hypothesis models have been reported to explain the formation mechanism of the complex of solidification structures^6–9^. These studies suggested that the convective transport of solute in the liquid phase is a very important factor in microstructural evolution. Therefore, it is necessary to develop a new method of externally manipulating fluid flow during solidification to enhance the impact of convection on solidification structures of peritectic alloys.

It is well known that static magnetic fields can damp fluid flows thereby promoting the development of crystal structures. However, the application of static magnetic fields during directional solidification can create some special phenomena. Moreau *et al*.^10^ found that the magnetic field caused the formation of segregation in directionally solidified Cu-Ag alloys. Lehmann *et al*.^11^ suggested that the magnetic field increased solute transport in the interdendritic region and decreased the dendrite spacing. Shen *et al*.^12^ found that the magnetic field modified the solidification morphology of Sn-Pn alloys during directional solidification. Kao *et al*.^13^ modeled the mechanism of magnetic field induced macrosegregation and dendritic refinement that demonstrated that static magnetic fields lead to a large scale flow circulation in the liquid phase. Experimental results have shown that the application of a static magnetic field during directional solidification could induce the formation of thermoelectric magnetic convection (TEMC)^14–17^. The initial driver for TEMC is indentified as Lorentz force in the liquid phase caused by the interaction of thermoelectric current and the magnetic field^18^. The thermoelectric current is generated by the diffusion of charge carriers in materials from the hot side to the cold side caused by temperature gradient. The definitive evidence of TEMC has been observed by means of *in situ* synchrotron X-ray radiography^19^. Although most works related to the effect of TEMC on microstructures have been investigated, these studies concerned with orientation and peritectic reaction in directionally solidified peritectic alloys are still missing. Therefore, TEMC under static magnetic fields provides opportunities to develop a novel method for extending the influence of convection on microstructural evolution in directionally solidified peritectic alloys.

In this work, Cu-10.5 at.% Sn is selected to investigate the crystal orientation of primary phase and the occurrence of peritectic reaction during directional solidification under a transverse static magnetic field. According to the Cu-Sn phase diagram, the phase transformation of Cu-10.5 at.% Sn starts with solidification of primary *α* phase from liquid phase (*L*). Then following peritectic reaction occurs at 1071 K, $L+\alpha \to \beta$. The primary *α* phase is a face-centered cubic structure, and the peritectic *β* phase is a body-centered cubic structure. Then following eutectoid reaction occurs at 859 K, $\beta \to \gamma +\alpha$. At 793 K, following eutectoid reaction occurs, $\gamma \to \alpha +\delta$. However, Liu *et al*.^20^ reinvestigated this system and found that the *β* and *γ* domains are effectively a continuous two-stage transition. These authors suggested that the $\beta \to \gamma +\alpha$ reaction is non-existent and the *β* and *γ* can be considered the same phase. Our preliminary results on the formation of band and island structures under the magnetic field in directionally solidified Fe-Ni and Pb-Bi peritectic alloys were reported earlier^15^. Subsequently, a systematic investigation on the orientation and peritectic reaction under the magnetic field in directionally solidified Cu-10.5 at.% Sn peritectic alloy was carried out to understand the effect of the magnetic field on the peritectic solidification process further.

## Description of the Experimental Device

Peritectic alloy Cu-10.5 at.% Sn was directionally solidified under various transverse static magnetic fields. The initial alloy was prepared from 99.99% pure Cu and Sn in a vacuum induction suspension-melting furnace. The cast sample was put into alumina tube with an inside diameter of 3 mm and length of 200 mm for directional solidification in a Bridgman crystal growth furnace equipped with a direct current transverse magnetic field device. The direct current transverse magnetic field device could produce continuously variable magnetic lines of force oriented perpendicular to the crystal growth direction. The Bridgman crystal growth furnace consisted of heat and cool controllers. A graphite-heated tube within an argon protection environment could heat the sample in the furnace. The temperature in the furnace could reach 1600 ± 1 °C and controlled by a Pt/6Rh-Pt/30Rh thermocouple. The cool controller was a water-cooled cylinder containing liquid Ga-In-Sn metal. The temperature gradient in the sample could be controlled by adjusting the temperature in the furnace heat zone, which was insulated from the liquid Ga-In-Sn metal by alumina ceramic disk. To perform directional solidification, the furnace was designed so that the cast sample in the tube moved downward through the heat zone into the liquid Ga-In-Sn metal. During the experiment, the cast sample in the tube was melted and then directionally solidified in the furnace by pulling the tube at various speeds while applying different magnetic field strengths. The directionally solidified sample was then etched, and the solidification structure obtained from the etched sample was examined by optical microscopy and scanning electron microscopy (SEM). Energy dispersive X-ray spectroscopy (EDS) and electronic backscatter diffraction (EBSD) were used to measure the solute content distributions and the crystal orientation characteristics, respectively.

## Experimental Results

**Figure 1:**
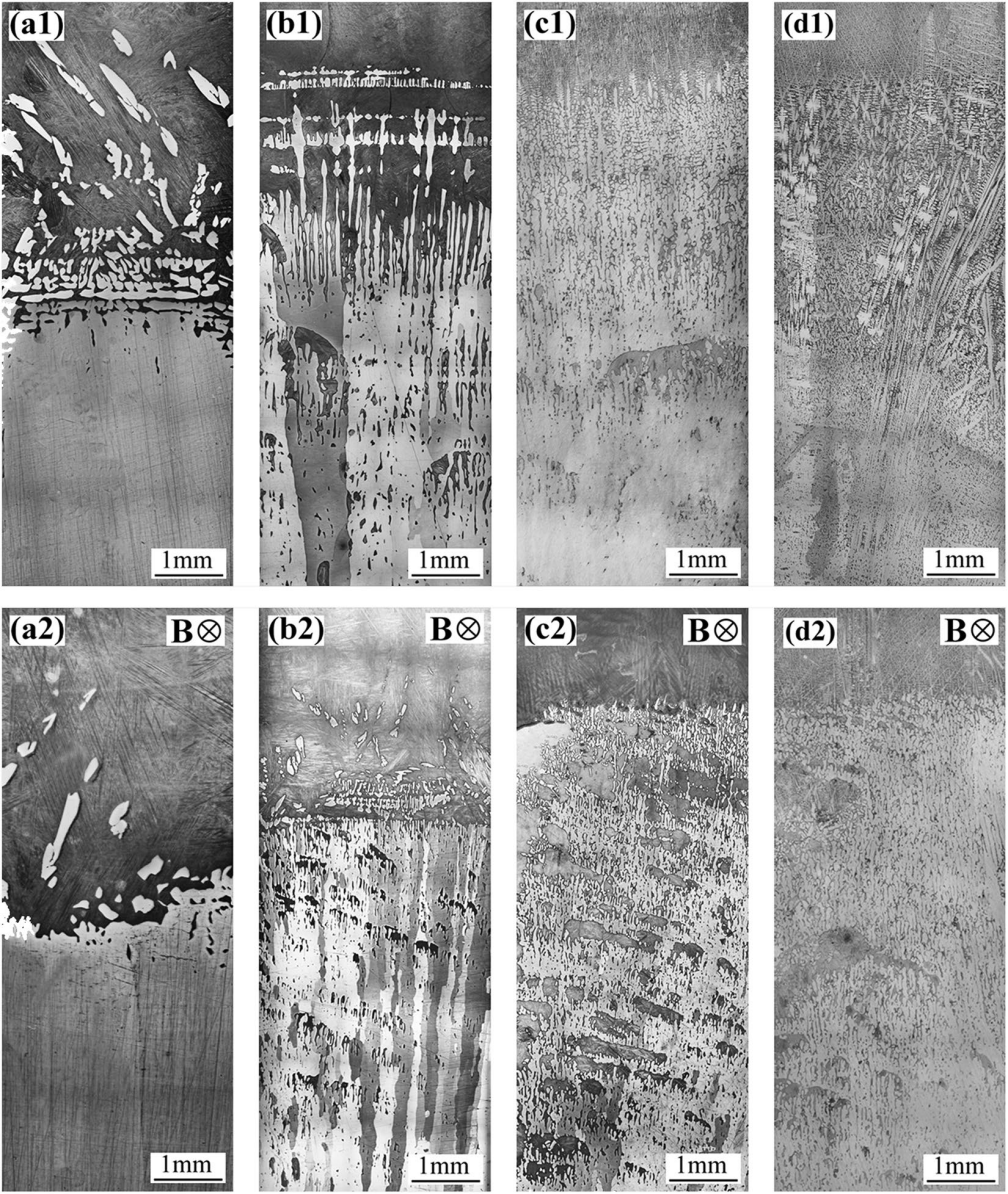
Solidification structures near the solid-liquid interface in peritectic alloy Cu-10.5 at.% Sn directionally solidified at a temperature gradient of 100 K/cm and various growth speeds without and with the application of a 0.7 T transverse static magnetic field: (**a**) 0.5 μm/s; (**b**) 2 μm/s; (**c**) 5 μm/s; (**d**) 50 μm/s.

Figure 1 shows the solidification structures near the solid-liquid interface in peritectic alloy Cu-10.5 at.% Sn directionally solidified at various growth speeds without and with the application of a 0.7 T transverse static magnetic field. The light and dark colors are primary *α* and liquid phases, respectively. Figure 1(a1) shows that primary *α*-island associated with the growth of primary *α*-cell front at 0.5 μm/s without the magnetic field. Figure 1(a2) shows that the application of a 0.7 T magnetic field at 0.5 μm/s demolished the growth interface, which implied that some macro-segregation is appeared, i.e. solute enrichment in the liquid phase ahead of the growth front of primary *α*-cell. Although a similar structure grew at 2 μm/s, as shown in Fig. 1(b1,b2), the imposition of a 0.7 T magnetic field at a higher growth speed weakened growth interface demolition and caused channel segregation formation. Fig. 1(c1,c2) show that the application of a 0.7 T magnetic field enhanced the channel segregation to form at 5 μm/s. However, applying a 0.7 T magnetic field at 50 μm/s had negligible impact on solidification structure, as shown in Fig. 1(d1,d2).

**Figure 2:**
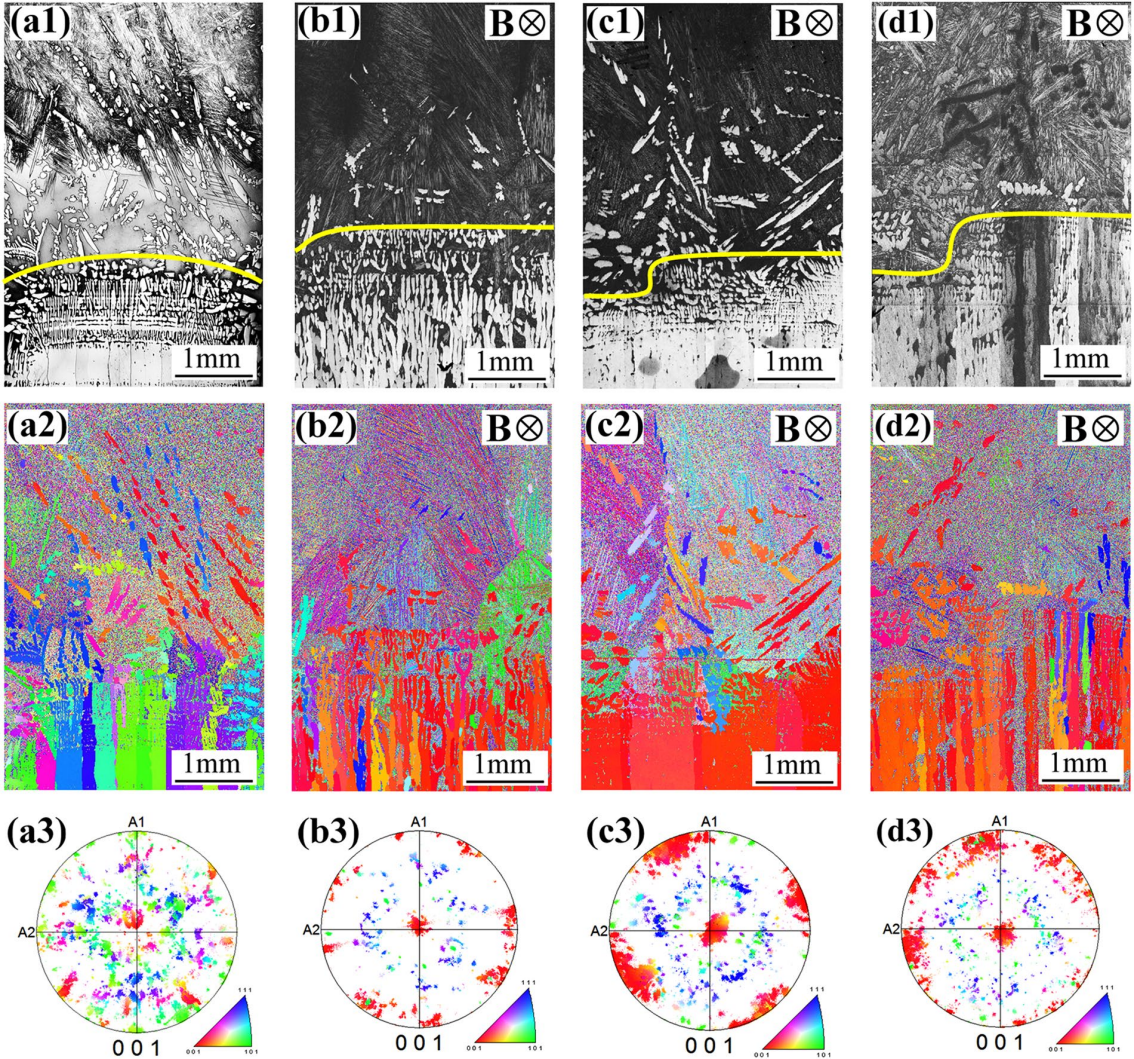
Solidification structures, and the corresponding EBSD maps and 〈001〉 pole figures for primary *α* phase in Cu-10.5 at.% Sn directionally solidified at a temperature gradient of 100 K/cm and growth speed of 1 μm/s under various magnetic fields: (**a**) 0 T; (**b**) 0.1 T; (**c**) 0.5 T; (**d**) 0.7 T.

Figure 2 shows the solidification structures, and the corresponding EBSD maps and 〈001〉 pole figures for primary *α* phase in Cu-10.5 at.% Sn directionally solidified at 1 μm/s under various transverse static magnetic field strengths. Yellow lines represent the schematic illustration of the macroscopic interface shape. Figure 2(a1) shows that a slightly convex primary *α-*cell structure grew on the macroscopic scale without the magnetic field. The application of a 0.1 T magnetic field demolished primary *α-*cell structure on one side of the sample and caused a sloping macroscopic interface shape, as shown in Figure 2(b1). Figure 2(c1) and (d1) show the solidification structures under 0.5 T and 0.7 T magnetic fields, respectively. The comparison of the macroscopic interface shape under various magnetic fields indicates that the magnetic field caused the sloping macroscopic interface shape formation and the amplitude increased with increase in the magnetic field strength. In addition, the application of the transverse static magnetic field modified the orientation of primary *α* phase during directional solidification. Figure 2(a2) and (a3) show the EBSD map and the corresponding 〈001〉 pole figure for primary *α* phase without the magnetic field, respectively. Different colors denote growth orientations, indicating that primary *α* phase region is mainly made of several solid cells with different colors, and each cell has the same color and thus the same crystal orientation. However, the application of a 0.1 T magnetic field caused the 〈001〉 crystal direction of primary *α*-cell began to move towards the solidification direction as shown in Fig. 2(b2,b3). With the increase of the magnetic field strength, the alignment of primary *α* phase enhanced. The above experimental results indicate that the imposition of the transverse static magnetic field demolished the growth interface of primary *α*-cell and caused the 〈001〉 crystal direction of primary *α* phase along solidification direction.

**Figure 3:**
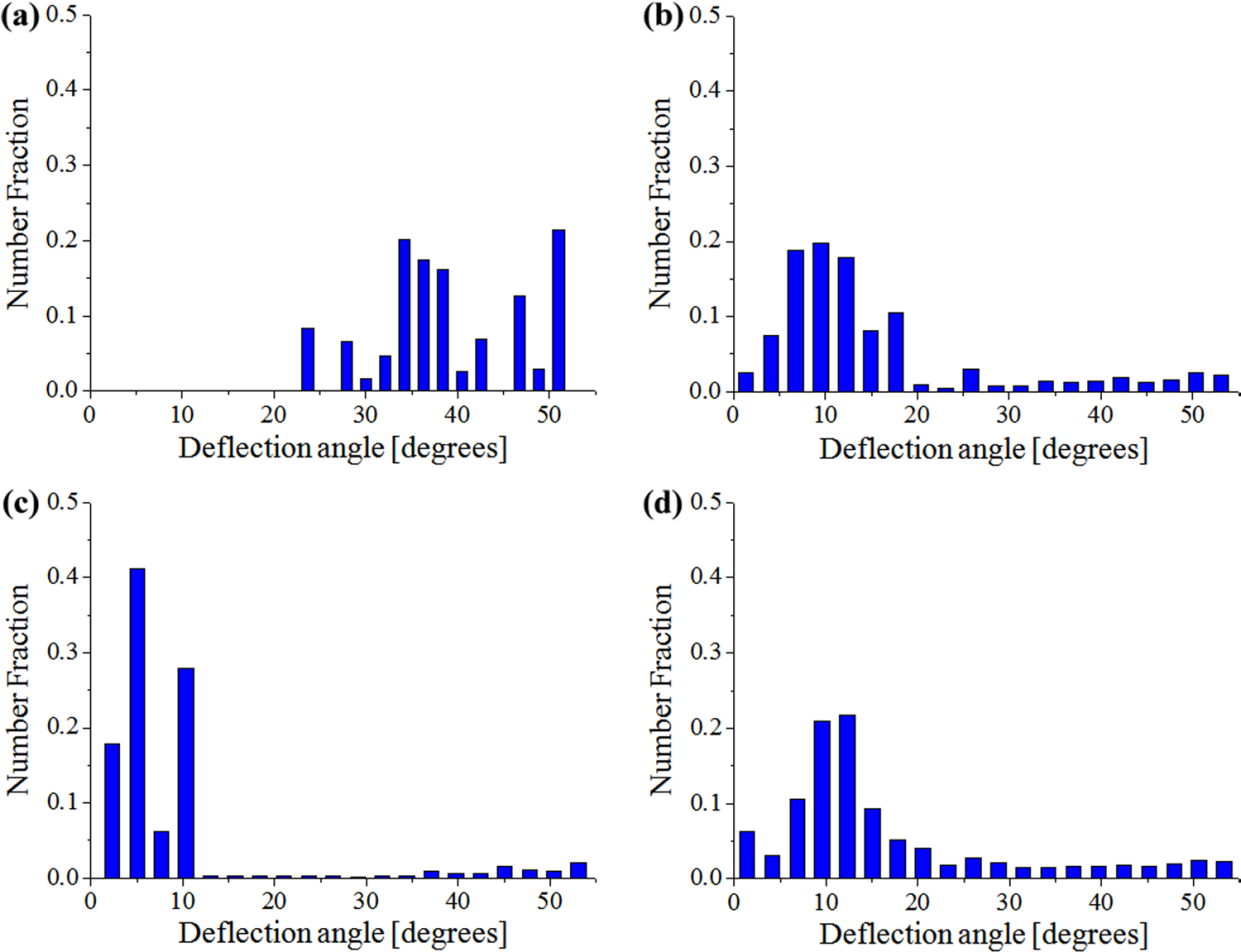
Number fraction of the 〈001〉 crystal direction of primary *α*-cell deviation from the solidification direction (corresponding to Fig. 2) under various magnetic fields: (**a**) 0 T; (**b**) 0.1 T; (**c**) 0.5 T; (**d**) 0.7 T.

Furthermore, number fraction of the 〈001〉 crystal direction of primary *α*-cell deviation from the solidification direction (corresponding to Fig. 2) was measured under various magnetic fields. As shown in Fig. 3(a), the deflection angle between the 〈001〉 crystal direction of primary *α*-cell and the solidification direction is more than 24 degree. Although the application of a 0.1 T magnetic field decreased the deflection angle to about 10 degree and alignment effect enhanced under a 0.5 T magnetic field, as shown in Fig. 3(b,c), a more intense magnetic field increased the deflection angle to about 10 degree again as shown in Fig. 3(d).

**Figure 4:**
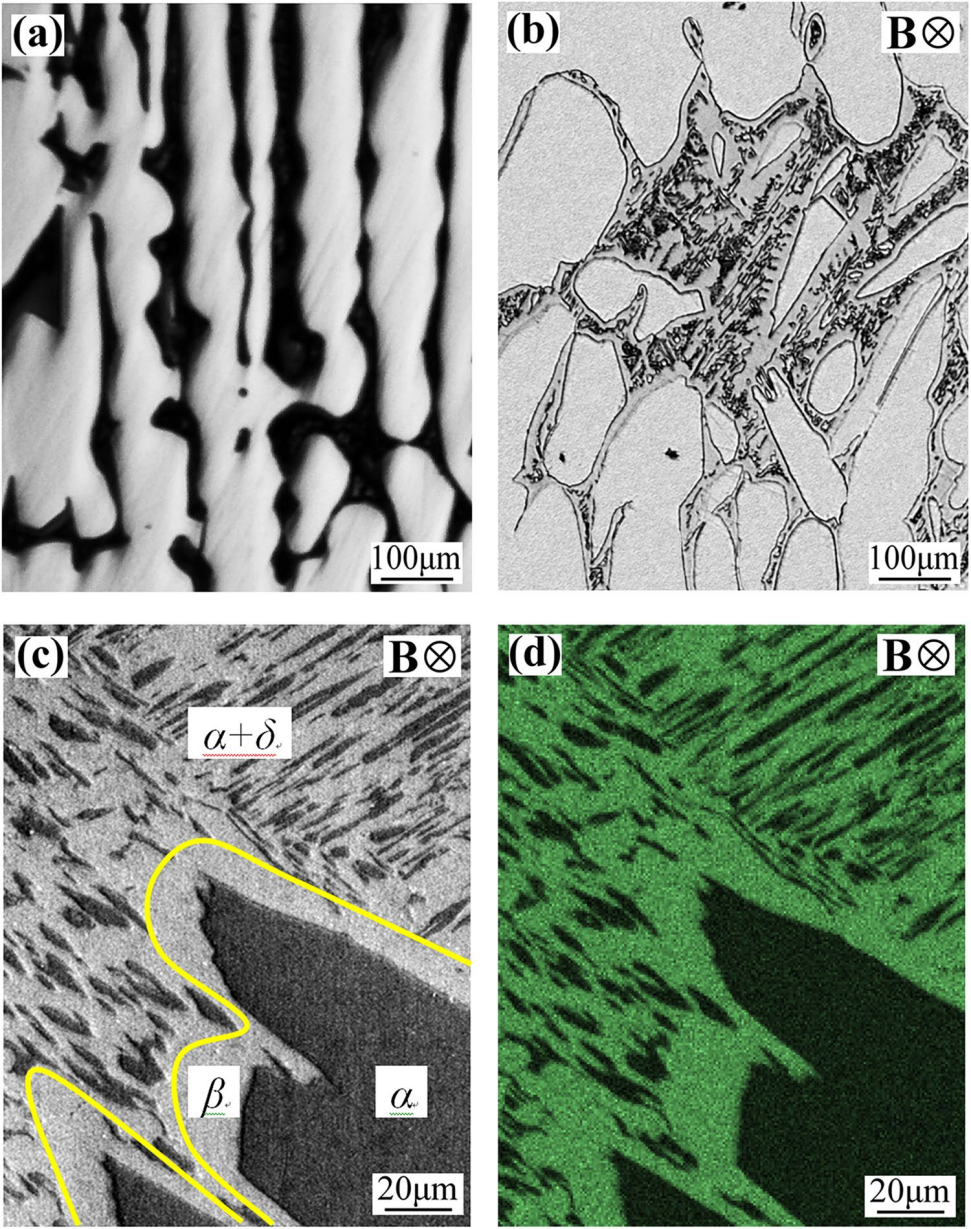
Microstructure in Cu-10.5 at.% Sn directionally solidified at a growth speed of 1 μm/s without and with a 0.3 T magnetic field: (**a**) 0 T; (**b**) 0.3 T; (**c**) and (**d**) show the SEM and the corresponding EDS map for the Sn solute content under a 0.3 T magnetic field, respectively.

Figure 4 shows the microstructure in Cu-10.5 at.% Sn directionally solidified at 1 μm/s without and with the application of a 0.3 T transverse static magnetic field, which caused the occurrence of peritectic reaction. As shown in Fig. 4(a,b), a thick layer of peritectic *β* phase surrounds the primary *α*-grains under the magnetic field. Owing to the eutectoid transformation, the remaining *β*-melt transformed into an (*α* + *δ*) eutectoid structure. Figure 4(c) and (d) show the SEM and the corresponding EDS map for the Sn solute content in Cu-10.5 at.% Sn directionally solidified under a 0.3 T magnetic field, respectively. The green and dark colors respectively imply the peritectic (high content) and primary (low content) phases, showing that the magnetic field induced the occurrence of peritectic reaction.

**Figure 5:**
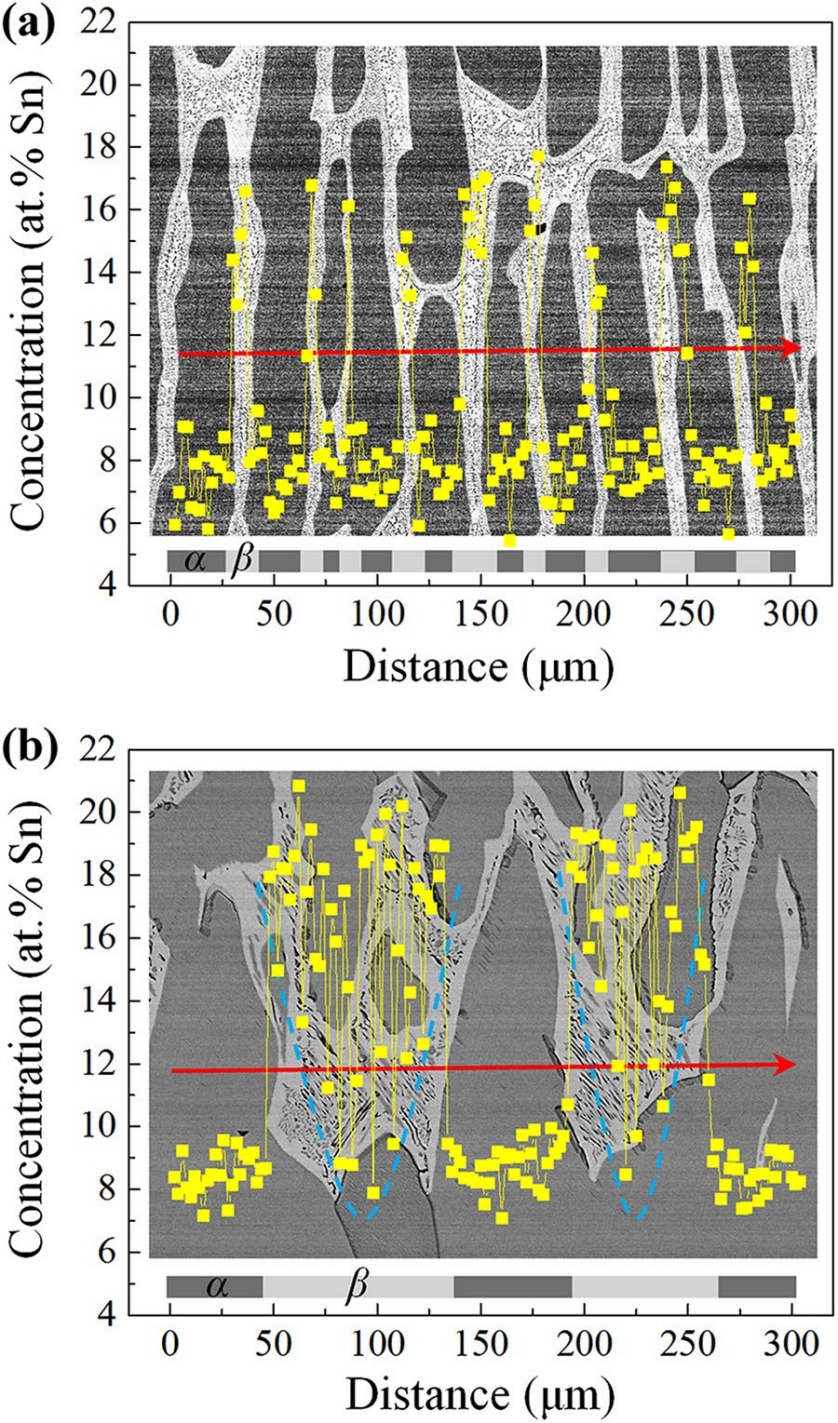
Radial distribution of Sn solute content between the primary *α*-cells along the red line for the sample in Cu-10.5 at.% Sn directionally solidified at a growth speed of 1 μm/s without and with a 0.3 T magnetic field: (**a**) 0 T; (**b**) 0.3 T.

Moreover, the distribution of Sn solute content in the interdendritic region was measured by EDS. Figure 5 shows the radial distribution of Sn solute content between the primary *α*-cells along the red line for the sample in Cu-10.5 at.% Sn directionally solidified at 1 μm/s without and with a 0.3 T magnetic field. Note that, due to the eutectoid transformation and the feature size of (*α* + *δ*) structure is less than the resolution limit of EDS, the (*α* + *δ*) structure could not be accurately indexed. The comparison of the samples without and with the magnetic field indicates that the magnetic field caused the periodic distribution of Sn solute content becomes long and irregular, and increased the maximum value of Sn content between the primary *α*-cells region. Although the magnetic field promoted solute transport thereby decreasing the dendrite spacing, the microstructural features under the magnetic field became coarser. This was probably because the occurrence of peritectic reaction under the magnetic field, which can demolish the growth of primary *α* phase.

## Computation Description and Results

Owing to the thermoelectric effect, thermoelectric current loops can be created around the solid-liquid interface when there is a temperature difference between solid and liquid phases. When the directions of thermoelectric currents are not parallel to those of magnetic fields, Lorentz forces can be produced by the combination of electric and magnetic fields. Moreover, Lorentz forces in the liquid further induce the generation of TEMC.

TEMC and the corresponding solute transport particle tracing under the transverse static magnetic field were numerically simulated in 3-D at the sample and 2-D at the liquid-*α*-*β* tri-junction. TEMC was firstly computed, when simulation came to steady state, solute particles evenly released in the liquid phase to calculate the effect of TEMC on solute transport. The model consisted of liquid- and solid- phase conductions, electric and magnetic field interactions, hydrodynamics, and melt particle tracings at controlled conditions of temperature gradient and magnetic field strength. The simulation was carried out for a given solid- and liquid- phase shape in peritectic Cu-10.5 at.% Sn directionally solidified under a transverse static magnetic field. Table 1 shows the peritectic Cu-10.5 at.% Sn physical parameters used during the numerical simulation.

**Table 1:** Peritectic Cu-10.5 at.% Sn physical parameters used during the numerical simulation.

Symbol	Unit	Value
Thermoelectric power for the solid, *S*_*L*_	V/K	5.23 × 10^−6^
Thermoelectric power for the liquid, *S*_*S*_	V/K	7.6 × 10^−6^
Electrical conductivity in the solid, *σ*_*S*_	(Ω·m)^−1^	9 × 10^6^
Electrical conductivity in the liquid, *σ*_*L*_	(Ω·m)^−1^	4 × 10^6^
Dynamic viscosity, *μ*	Pa·s	1.25 × 10^−3^
Temperature gradient, *G*	K/cm	100
Diffusion coefficient, *D*	m^2^/s	5 × 10^−9^
Liquid composition at peritectic reaction, *C*_*L*_	wt.%	25.5
Liquidus slop of *α*, ${m}_{L}^{\alpha }$	K/wt.%	−11.03
Liquidus slop of *β*, `	K/wt.%	−5.53

These simulations were based on the thermoelectric effect that a temperature difference produces thermoelectric currents. The basic equation of the thermoelectric currents under the magnetic field can be constructed as:

1 $${j}\,=\,{\sigma }({u}\times {B})-{\sigma }\mathrm{SG}$$

2 $$\nabla \cdot {j}={\rm{0}}$$

where *j* is the thermoelectric current density, *σ* is the electrical conductivity, *u* is the fluid flow velocity, *B* is the magnetic field intensity, *S* is the thermoelectric coefficient, and *G* is the temperature gradient.

TEMC is governed by the Navier-Stokes equation, which for TEMC under the magnetic field can be written as:

3 $${\rho }\frac{\partial {u}}{\partial {t}}+{\rho }({u}\cdot \nabla {u})=-\,\nabla {p}+{j}\times {B}+{\rho }{\mu }{\nabla }^{2}{u}$$

where *p* is pressure, *ρ* is density, and *μ* is dynamic viscosity.

TEMC-induced the order of magnitude of the drag force acted on the solute transport in the liquid phase is

4 $${\rm{F}}=\frac{m{\mu }}{{\rho }d}({u}-{v})$$

where *F* is the drag force, *m* is the solute particle mass, *d* is the solute particle radius, and *v* is the solute particle velocity.

The numerical results are included in this paper for illustration the formation of TEMC and its effect on solute transport during directional solidification under the magnetic field only, a more detailed description of the basic assumptions and boundary conditions can be found in ref.^21^.

**Figure 6:**
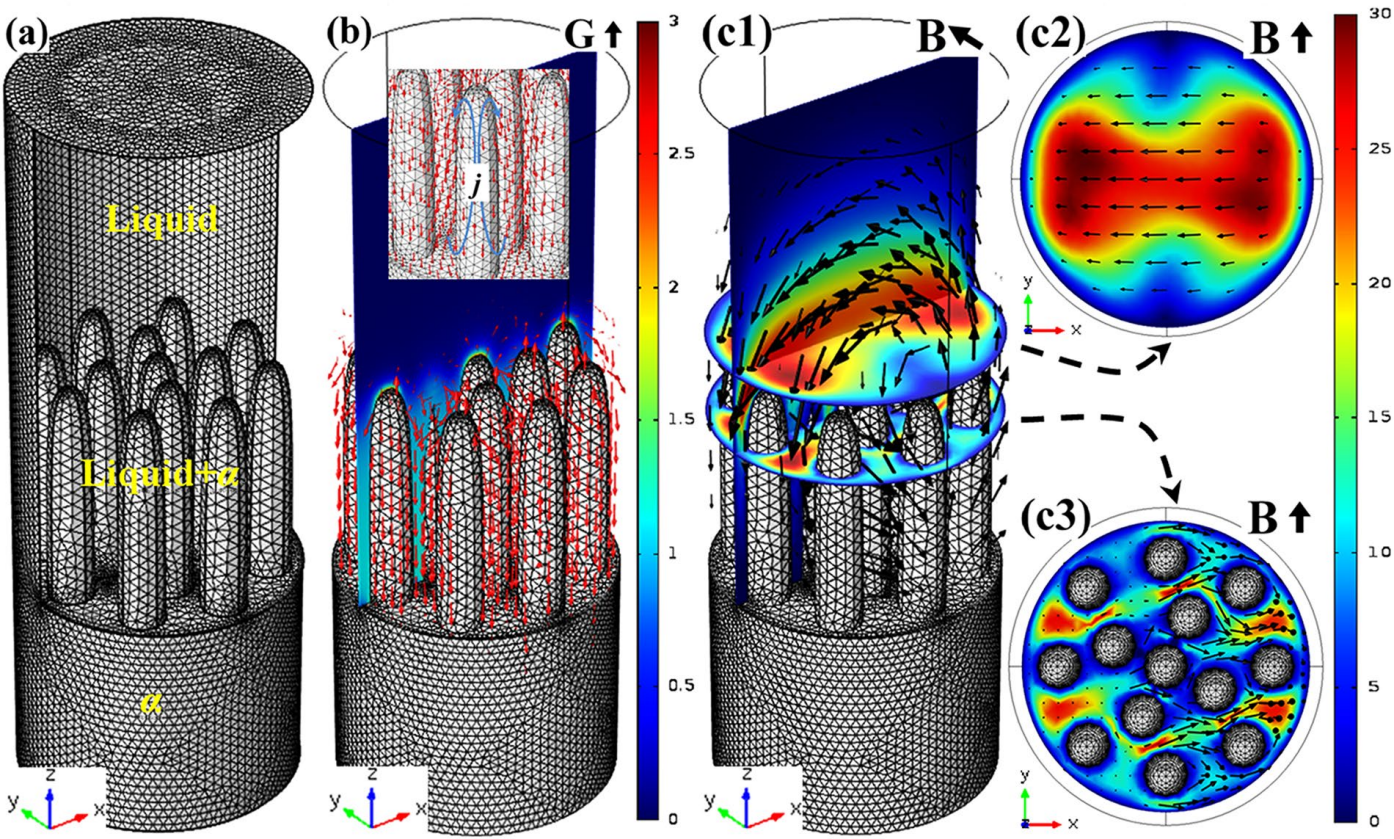
3-D numerical simulation of TEMC in Cu-10.5 at.% Sn directionally solidified under a 0.1 T magnetic field: (**a**) the geometry model used for the simulation; (**b**) the computed thermoelectric current (×10^6^ A/m^2^); (c1–c3) the computed TEMC (mm/s) in the liquid phase viewed from different axes.

**Figure 7:**
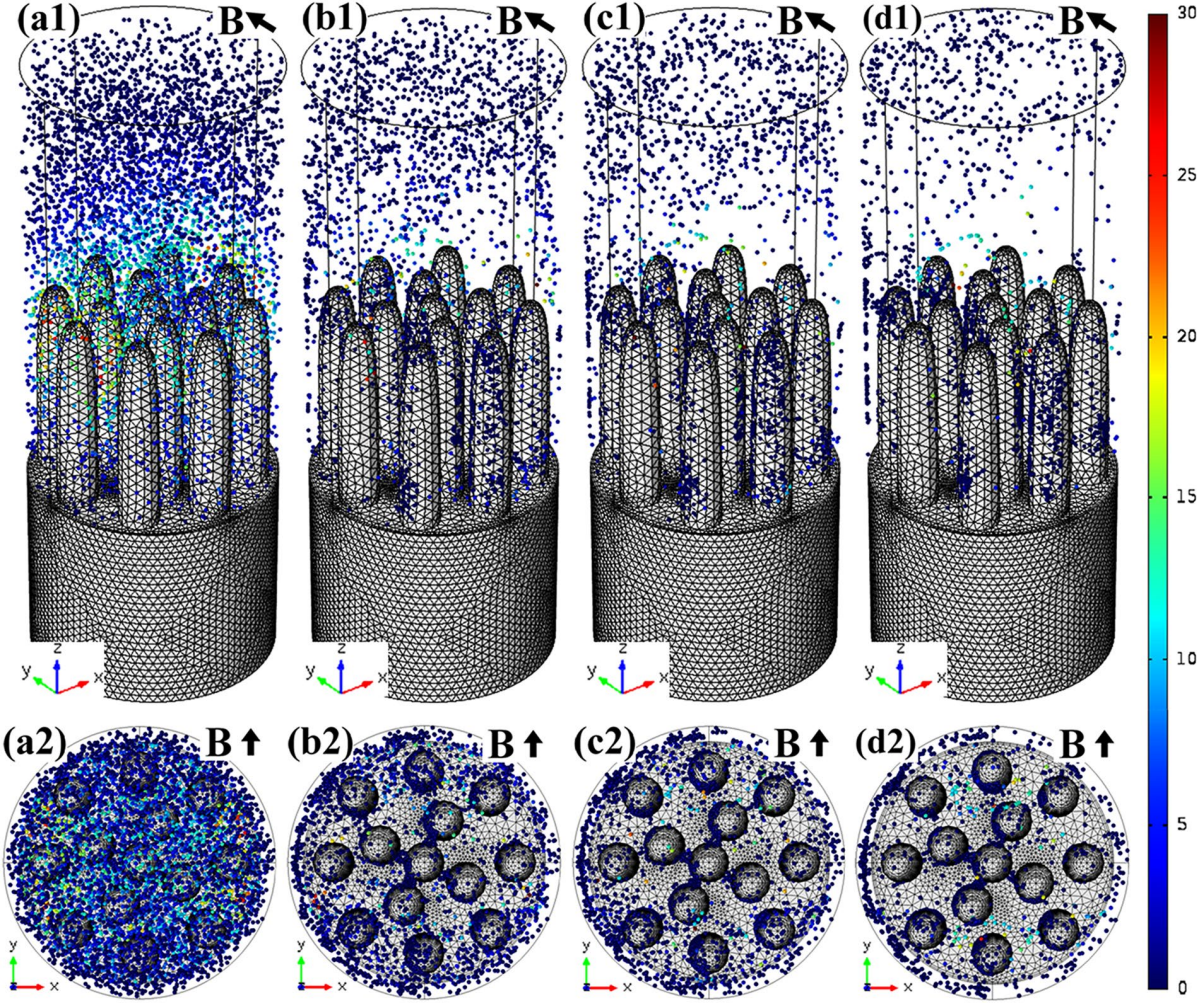
TEMC-induced solute transport particle tracing at different time levels in Cu-10.5 at.% Sn directionally solidified under a 0.1 T magnetic field: (**a**) 0.1 s; (**b**) 50 s; (**c**) 200 s; (**d**) 300 s.

**Figure 8:**
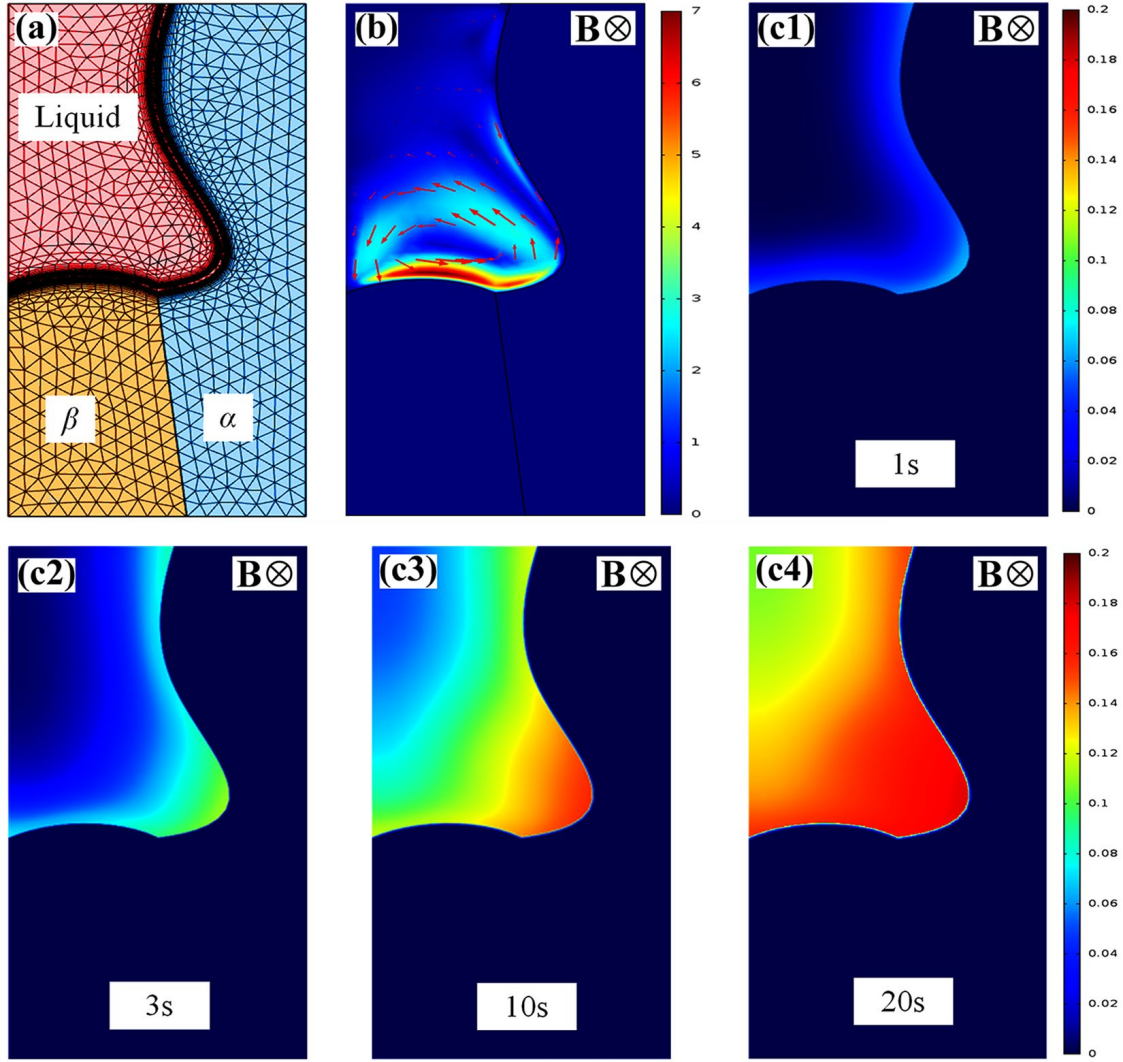
2-D numerical simulation of TEMC and its effect on the solute concentration around the liquid-*α*-*β* tri-junction in Cu-10.5 at.% Sn directionally solidified under a 0.1 T magnetic field: (**a**) the geometry model; (**b**) the computed TEMC (mm/s) in the liquid phase; (c1–c4) show the effect of TEMC on solute concentration in the liquid phase at different time levels under a 0.1 T magnetic field.

TEMC under the transverse static magnetic field and its effect on solute transport in 3-D at the sample and 2-D at the liquid-*α*-*β* tri-junction during directional solidification were investigated in detail. Figure 6 shows the 3-D numerical simulation of TEMC in Cu-10.5 at.% Sn directionally solidified under a 0.1 T magnetic field. Figure 6(a) shows the geometry model used for the simulation. The cylinder is regarded as primary *α*-phase, and the spacing of these cylinders correspond to primary arm spacing. Figure 6(b) shows the computed thermoelectric current (×10^6^ A/m^2^) forming endless loops (i.e., the red arrows) around *α*-cells at a temperature gradient of 100 K/cm. Figure 6(c1–c3) show the computed TEMC (mm/s) in the liquid viewed from different axes. The arrows and colors respectively imply the direction and velocity of TEMC, indicating that unidirectional TEMC formed in the interdendritic regions from one side of the sample to the other, and then returned from the higher liquid phase. Figure 7 shows TEMC-induced solute transport particle tracing at different time levels in Cu-10.5 at.% Sn directionally solidified under a 0.1 T magnetic field. Particles and colors represent the solute and corresponding velocity, respectively. It can be found that TEMC drove solute transport thereby overlapping the solute particles, indicating that TEMC increased solute concentration in the interdendritic regions. With the increase of the time levels, the influence of TEMC on solute concentration increased. Furthermore, in order to clarify the effect of TEMC on the solute concentration around the liquid-*α*-*β* tri-junction, 2-D numerical simulation was performed. Figure 8(a) and (b) show the geometry model and TEMC around the liquid-α-β tri-junction in Cu-10.5 at.% Sn directionally solidified under a 0.1 T magnetic field, respectively. Unidirectional TEMC formed from the front of *β* phase to *α* phase and returned to the above liquid. Figure 8(c1–c4) show the effect of TEMC on solute concentration around the liquid-*α*-*β* tri-junction at different time levels under a 0.1 T magnetic field. The colors represent the solute concentration difference and the numbers in the color bar show the significance of maximum solute enrichment, indicating that TEMC caused solute enrichment thereby increasing the solute concentration at the liquid-*α*-*β* tri-junction and enhanced over time.

## Discussion

### Transverse static magnetic field induced orientation of primary phase

**Figure 9:**
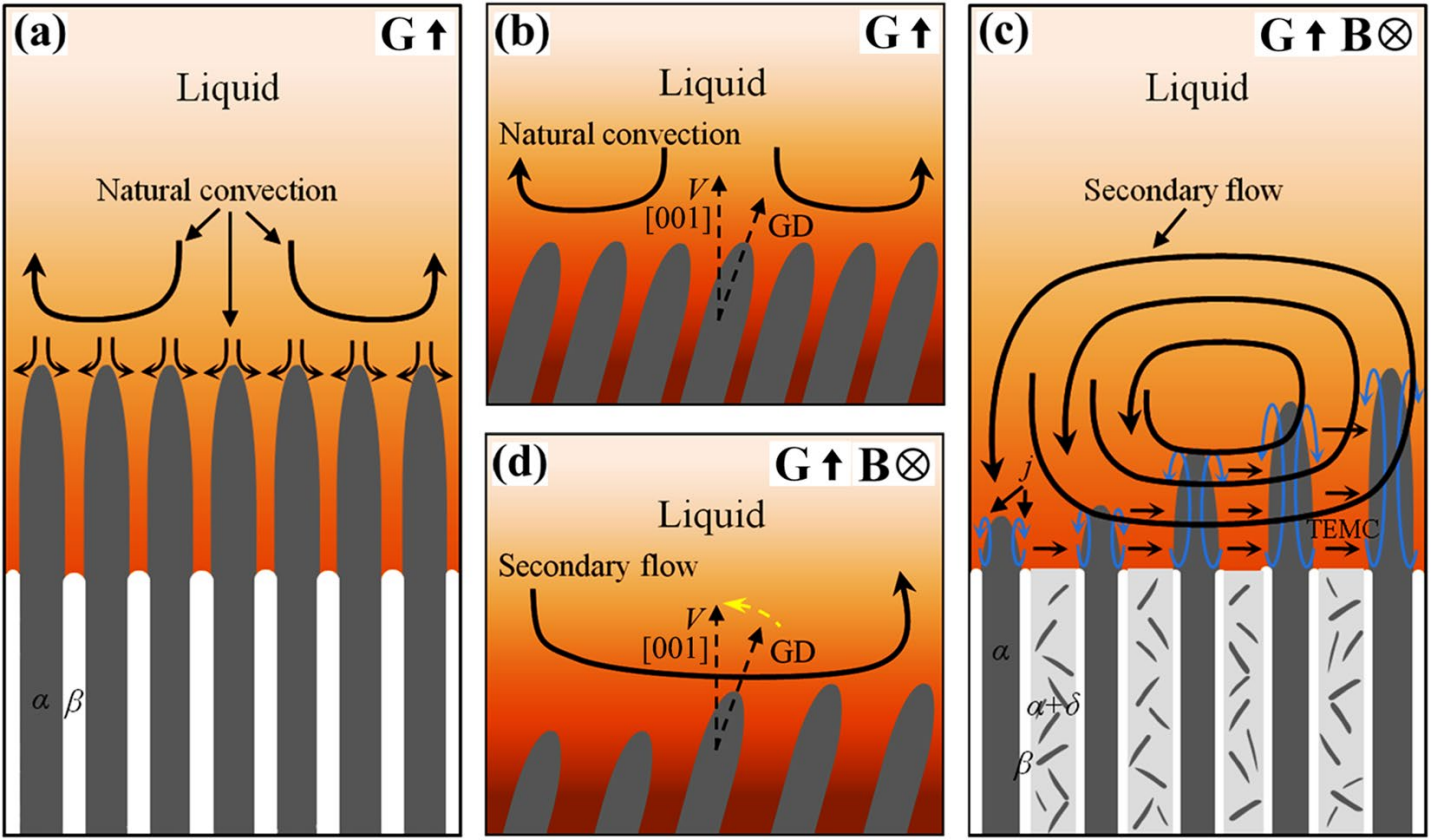
Schematic diagram of the effect of TEMC on the microstructure and crystal orientation in Cu-10.5 at.% Sn directionally solidified under a transverse static magnetic field: (**a**) the natural convection near the solid-liquid interface without the magnetic field; (**b**) the growth direction (GD) of primary *α* phase at the natural convection condition; (**c**) TEMC under the magnetic field and its effect on the microstructure; (**d**) the crystal orientation of primary *α* phase by the secondary flow caused by TEMC.

The above experimental results reveal that the imposition of a transverse static magnetic field in Cu-10.5 at.% Sn directionally solidified caused the 〈001〉 crystal direction of primary *α* phase along the solidification direction (see Fig. 2). Although interphase anisotropy, fluid flows and solute transports in the interdendritic region can affect the crystal orientation in solid phase^22,23^, primary *α* and peritectic *β* phases belong to the cubic crystal system. Therefore, for Cu-10.5 at.% Sn directionally solidified under the magnetic field, the growth direction of crystal can be determined by the preferred growth orientation and the heat flow direction. When the two directions are not identical, the crystal grows along the direction of heat flow at low growth speeds during directional solidification. With the increase of growth speed, the crystal growth direction modifies from the heat flow direction to the preferred growth orientation. If the direction of heat flow is the same as the preferred growth orientation, the crystal growth direction turns to the solidification direction as the velocity of heat flow increases. Therefore, the formation of TEMC under the magnetic field should be responsible for the 〈001〉 crystal direction of primary *α* phase along the solidification direction during directional solidification. Figure 9 shows a schematic diagram of the effect of TEMC on the microstructure and crystal orientation in Cu-10.5 at.% Sn directionally solidified under a transverse static magnetic field. As shown in Fig. 9(a), in the case of no magnetic field, the rejection of lighter Sn solute at the solidifying front clearly induces the formation of natural convection during directional solidification. The natural convection causes symmetry in the distribution of solute concentration and temperature, which results in the growth of columnar cells or dendrites deviation from the solidification direction. It has been proved that *α* phase is face-centered cubic structure, indicating that the 〈001〉 crystal direction of *α* phase may be the preferred growth orientation. During axial directional solidification, the crystal will grow along the solidification direction (*i.e*. the direction of [001]). Thus, the formation of natural convection causes the growth of primary *α* phase deviates from the solidification direction during directional solidification (see Fig. 9(b)). When a transverse static magnetic field is imposed during directional solidification, unidirectional TEMC formed in the liquid phase between the primary *α*-cells from one side of the sample to the other. Unidirectional TEMC can induce a secondary circulation flow in the liquid phase ahead of the growth front of solid, as shown in Fig. 9(c). According to the numerical simulation results, TEMC and the corresponding secondary flow can drive solute transport through its flow thereby concentrating solute density on one side of the sample. Such solute enrichment results in decreases in interface temperature and the deformation of solidification interface morphology. When the concentration of solute attains to a critical level, macrosegregation appears at one side of the sample and the sloping macroscopic interface shape forms. In addition, TEMC and the corresponding secondary flow can also drive heat transfer through its flow, thereby increasing the velocity of heat flow in the direction of solidification (the same as preferred growth orientation of primary *α* phase). With the increase of TEMC velocity, the crystal growth direction turns to the solidification direction as shown in Fig. 9(d). Our previous studies suggested that TEMC velocity was increased first and then decreased with the increase of magnetic field intensity^15^. This was responsible for the average deflection angle increased when the magnetic field increased from 0.5 T to 0.7 T, as shown in Fig. 3(d). Therefore, the deformation of macroscopic interface morphology of primary *α*-cells and the modification of the crystal orientation of primary *α* phase in Cu-10.5 at.% Sn directionally solidified under the magnetic field should be attributed to TEMC-driven solute transport and heat transfer, respectively.

### Transverse static magnetic field induced occurrence of peritectic reaction

The above experimental results also indicate that the application of a transverse static magnetic field in Cu-10.5 at.% Sn directionally solidified enhanced the occurrence of peritectic reaction (see Fig. 4). During directional solidification of Cu-Sn peritectic system, the rejection of Sn solute at the solidifying front will change the local composition. Since the solidification temperature decreases with the increase in Sn solute content, the solidification temperature for the Sn-rich melt around the primary *α* phase decreases. When the Sn solute reaches suitably concentrated and the solidification temperature decreases to peritectic reaction temperature, the peritectic *β* phase starts to nucleate and grow through the peritectic reaction. During directional solidification, the peritectic reaction can be described by the maximum growth speed and the minimum undercooling laws suggested by Fredriksson and Nylén^24^. They assumed that the peritectic *β* phase is a flat plate-like layer covering the primary *α* phase. The thickness of peritectic *β*-phase from the peritectic reaction is given by

5 $${h}=\frac{9{D}}{8{\rm{\pi }}{V}}{{\rm{\Omega }}}^{2}{({\rm{1}}-\frac{2}{\pi }{\rm{\Omega }}-\frac{1}{2{\rm{\pi }}}{{\rm{\Omega }}}^{2})}^{-{\rm{2}}}$$

6 $${\rm{\Omega }}=\frac{{{C}}_{{L}}^{{\alpha }}-{{C}}_{{L}}^{{\beta }}}{{{C}}_{{L}}^{{\beta }}-{{C}}_{{\beta }}^{{L}}}$$

where, *D* is the diffusion coefficient of solute in the liquid phase, Ω is the supersaturation, *V* is the growth speed, and ${C}_{i}^{j}$ are the interface concentrations. In order to simplify calculation, linearization of the peritectic phase diagram is used by setting

7 $${{C}}_{{L}}^{{i}}={{C}}_{{L}}-\frac{{\rm{\Delta }}{T}}{{{m}}_{{L}}^{{i}}}{\rm{i}}=\alpha \,{\rm{or}}\,\beta $$

where *C*_*L*_ is the liquid composition at peritectic reaction, Δ*T* is the constitutional supercooling due to Sn solute enrichment, ${m}_{L}^{i}$ is the liquidus slop of *i* phase.

As a result, the supersaturation in Cu-Sn peritectic system can be defined as

8 $${\rm{\Omega }}=\frac{3{\rm{\Delta }}{T}}{\mathrm{120}+\mathrm{10}{\rm{\Delta }}{T}}$$

Buoyancy-driven flow model shows that the interdendritic constitutional supercooling increases with the increase in the solute transport rate^25^. Under the transverse static magnetic field, TEMC and the corresponding secondary flow will be produced in the liquid phase during directional solidification, as shown in Fig. 6. These flows will enhance the solute transport (see Figs 7 and 8) and then increase the interdendritic constitutional supercooling. According to Equations 5 and 8, TEMC and the corresponding secondary flow increased the interdendritic constitutional supercooling and promoted the formation of peritectic *β* phase. Moreover, the local solidification time (*t*) also affects the formation of peritectic *β* phase. During directional solidification, *t* can be estimated as $t=\Delta {T}/{GV}$. It is well known that the thickness of peritectic phase enhances with the increase in the local solidification time^1^. The convection under the magnetic field increases *t* through improving the local constitutional supercooling by increasing solute transport. Therefore, TEMC-driven solute transport should be responsible for the occurrence of peritectic reaction under the transverse static magnetic field.

## Conclusions

The modification of microstructure and crystal orientation in Cu-10.5 at.% Sn directionally solidified at a temperature gradient of 100 K/cm under various transverse static magnetic fields (up to 1 T) has been investigated. The main results and conclusions are summarized as follows:

1. The imposition of the magnetic field demolished the macroscopic interface morphology and caused the 〈001〉 crystal direction of primary *α* phase along the solidification direction.
2. The application of the magnetic field enhanced the occurrence of peritectic reaction.
3. Numerical simulation results indicate that the formation of TEMC under the magnetic field drives solute transport and induces solute enrichment in the interdendritic region.
4. The 〈001〉 crystal direction of primary *α* phase became oriented along the solidification direction, the deformation of macroscopic interface morphology and the occurrence of peritectic reaction should be attributed to TEMC-driven heat transfer and solute transport, respectively.
